# Supplementary material for: Whole-genome analysis of Escherichia coli isolated from wild Amur tiger (Panthera tigris altaica) and North China leopard (Panthera pardus japonensis)
Source: PeerJ. 2024 May 6;12:e17381. doi: 10.7717/peerj.17381 (PMC11080988; doi:10.7717/peerj.17381)
Supplement: Supplemental Information 5 [file peerj-12-17381-s005.docx]

Dear Editors and Reviewers:

Subject: Author change request for the manuscript titled "Whole-genome analysis of *Escherichia coli* isolated from wild Amur tiger (*Panthera tigris altaica*) and North China leopard (*Panthera pardus japonensis*)" (Manuscript ID: 95486)

I hope this message finds you well. I am writing to request a change in the authorship for our manuscript entitled "Whole-genome analysis *Escherichia coli* isolated from wild Amur tiger (*Panthera tigris altaica*) and North China leopard (*Panthera pardus japonensis*)", which was submitted to PeerJ on January 2024. The manuscript ID is 95486.

We propose adding Denghui Chen as an author due to his comprehensive and critical contributions to the phenotypic antimicrobial drug susceptibility testing, a study conducted following the recommendations of a reviewer and entirely executed by him. His specific contributions are as follows:

1. Designing the phenotypic antimicrobial drug susceptibility testing: Denghui Chen significantly contributed to the planning phase of the experiment, which was conducted from March 1st to March 3rd. His responsibilities included the preparation of experimental apparatus, selection of appropriate antimicrobial medications, and preparation of bacterial strains.

2. Conducting the Laboratory Work: He was actively engaged in the laboratory work from March 4th to March 7th following the initial design. This phase involved the preparation of culture media, revival of bacterial cultures, and meticulous handling of the antimicrobial drug.

3. Analyzing the Resulting Data: Denghui Chen took the lead in organizing and analyzing the experimental results from March 8th to March 9th. His work included statistical analysis of the inhibition zones and comprehensive recording and interpretation of the collected data.

These contributions were critical to the success of our antibiotic resistance study component. The revised author list would be as follows:

Hongjia Li^1,^*, Tianming Lan^1,^*, Hao Zhai^2^, Mengchao Zhou^1^, Denghui Chen^1^, Yaxian Lu^1^, Lei Han^1^, Jinpu Wei^1^, Shaochun Zhou^3^, Haitao Xu^4^, Lihong Tian^1^, Guangshun Jiang^1^, Zhijun Hou^1^

1 College of Wildlife and Protected Area, Northeast Forestry University, Harbin, China

2 Ningxia Forestry Project Management Center, Yinchuan, China

3 Heilongjiang Research Institute of Wildlife, Harbin, China

4 Heilongjiang Siberian Tiger Park, Harbin, China

Corresponding Author:

Zhijun Hou^1^

College of Wildlife and Protected Area, Northeast Forestry University, Harbin, China

Email address: houzhijundb@163.com

Co-corresponding Author:

Guangshun Jiang^1^

College of Wildlife and Protected Area, Northeast Forestry University, Harbin, China

Email address: jgshun@126.com

* These authors contributed equally to this work.

All authors, including Denghui Chen, have been informed of this change and have agreed to it. We have also confirmed that this change complies with the journal's authorship criteria.

We apologize for any inconvenience this change may cause and kindly request your approval for this authorship modification.

Thank you for your time and consideration.

Sincerely,

Zhijun Hou

Northeast Forestry University
